# Optic Disc - Fovea Angle: The Beijing Eye Study 2011

**DOI:** 10.1371/journal.pone.0141771

**Published:** 2015-11-06

**Authors:** Rahul A. Jonas, Ya Xing Wang, Hua Yang, Jian Jun Li, Liang Xu, Songhomitra Panda-Jonas, Jost B. Jonas

**Affiliations:** 1 Beijing Institute of Ophthalmology, Beijing Tongren Eye Center, Beijing Tongren Hospital, Capital Medical University, Beijing Ophthalmology and Visual Science Key Lab, Beijing, China; 2 Department of Ophthalmology, Medical Faculty Mannheim of the Ruprecht-Karls-University of Heidelberg, Mannheim, Germany; University of Houston, UNITED STATES

## Abstract

**Purpose:**

To determine the optic disc-fovea angle (defined as angle between the horizontal and the line between the optic disc center and the fovea) and to assess its relationships with ocular and systemic parameters.

**Methods:**

The population-based cross-sectional Beijing Eye Study 2011 included 3468 individuals. A detailed ophthalmic examination was carried out. Using fundus photographs, we measured the disc-fovea angle.

**Results:**

Readable fundus photographs were available for 6043 eyes of 3052 (88.0%) individuals with a mean age of 63.6±9.3 years (range: 50–91 years) and a mean axial length of 23.2±1.0 mm (range: 18.96–28.87 mm). Mean disc-fovea angle was 7.76 ± 3.63° (median: 7.65°; range: -6.3° to 28.9°). The mean inter-eye difference was 4.01 ± 2.94° (median: 3.49°; range: 0.00–22.3°). In multivariate analysis, larger disc-fovea angle was associated (regression coefficient r^2^: 0.08) with older age (*P* = 0.009; standardized regression coefficient beta: 0.05), thinner RNFL in the nasal superior sector (*P*<0.001; beta: -0.17), superior sector (*P*<0.001; beta: -0.10) and temporal superior sector (*P*<0.001; beta: -0.11) and thicker RNFL in the inferior sector (*P*<001; beta: 0.13), nasal inferior sector (*P*<001; beta: 0.13) and nasal sector (*P* = 0.007; beta: 0.06), higher prevalence of retinal vein occlusion (*P* = 0.02; beta: 0.04), and with larger cylindrical refractive error (*P* = 0.04; beta: 0.04).

**Conclusions:**

The optic disc-fovea angle markedly influences the regional distribution of the RNFL thickness pattern. The disc-fovea angle may routinely be taken into account in the morphological glaucoma diagnosis and in the assessment of structure-function relationship in optic nerve diseases. Future studies may address potential associations between a larger disc-fovea angle and retinal vein occlusions and between the disc-fovea angle and the neuroretinal rim shape.

## Introduction

The angle between the optic disc center and the foveola (“disc-fovea angle”) is a landmark parameter of the posterior fundus, since, together with the disc-fovea distance, it characterizes the position of the optic nerve head in relationship to the foveola [1–5]. Since the retinal nerve fiber layer (RNFL) is centered on the optic nerve head and since the visual field and other psychophysical examinations are centered on the foveola, the disc-fovea angle influences the structure-function relationship in any optic nerve disease, in particular in glaucoma [6–17]. The structure-function relationship describes the association between psychophysical deficits, e.g., perimetric defects, and structural changes such as retinal nerve fiber layer defects [18–20]. The disc-fovea angle has been used to estimate the amount of ocular torsion [21–23]. It has been discussed whether the physiological postnatal growth of the globe influences the disc-fovea angle. In particular, it has remained elusive whether the marked changes in scleral thickness occurring with myopic axial elongation at the posterior fundus pole and whether the development of myopic maculopathy including the development of secondary macular Bruch’s membrane defects are associated with a change in the disc-fovea angle [24,25]. Since most of the previous studies had a hospital-based recruitment of study participants, were relatively small-scaled and did not examine a large array of other factors potentially associated with the disc-fovea angle, we conducted this study to measure the disc-fovea angle in a relatively large group of study participants who underwent a comprehensive ophthalmologic and general examination. To avoid the risk of a potential bias inherent to any hospital-based study, we chose the design of a population-investigation.

## Methods

The Beijing Eye Study 2011 is a population-based cross-sectional survey performed in Northern China and which has been described in detail previously [26,27]. The Medical Ethics Committee of the Beijing Tongren Hospital approved the study protocol and all participants gave informed written consent. Out of 4403 eligible individuals fulfilling the only inclusion criterion of an age of 50+ years, 3468 (78.8%) individuals (1963 (56.6%) women) participated. The mean age was 64.6 ± 9.8 years (median, 64 years; range, 50–93 years).

All participants underwent a structured questionnaire, systemic examinations, and a comprehensive ophthalmic examination. The latter included measurement of visual acuity, slit lamp examination of the anterior and posterior segment of the eye, and digital photography of the cornea, lens, macula and optic disc and fundus photography (fundus camera Type CR6-45NM; Canon Inc., Tokyo, Japan). Spectral domain optical coherence tomography (SD-OCT, Spectralis^®^, Heidelberg Engineering Co., Heidelberg, Germany), also with the enhanced depth imaging modality, was performed after pupil dilation to measure the thickness of the RNFL and of the subfoveal choroid [28]. The degree of cataract was determined using the standardized lens photographs as described recently [29]. Diabetic retinopathy was diagnosed on the fundus photographs [30]. Using the fundus photographs, we also measured the distance between the optic disc center and the foveola and the angle between the disc-fovea line and the horizontal. If the foveola was located above the horizontal optic disc axis, the angle measurement was noted as negative value. The technique of assessing the disc-fovea angle has already been described and applied in previous investigations by Lamparter and colleagues, Denniss and associates, Choi and coworkers, and others [8,12,14,17]. To obtain the disc-fovea distance in real measurements, we corrected the magnification by the optic media of the eye and by the fundus camera using the Littmann method [31].

Statistical analysis was performed using a commercially available statistical software package (SPSS for Windows, version 22.0, IBM-SPSS, Chicago, IL, USA). For the inter-individual comparisons, only eye selected per subject was included into the statistical analysis. For the intra-individual inter-eye comparison, both eyes of the individuals were taken into account. For the assessment of the inter-eye difference in the disc-fovea angle, we used the measurements obtained in both eyes of the same individual and calculated the difference in the value of right eye minus left eye and reported the absolute value of the difference. In a first step of the statistical analysis, we examined the distribution of the disc-fovea angle using the Kolmogorov-Smirnov test and we calculated the mean ± standard deviations of the parameter. In a second step of the analysis, we analyzed the associations between the disc-fovea angle and one of any other ocular and systemic variables by linear regression. If in this univariate analysis a binary variable was tested, we applied the Student t-test. In a third step, we conducted a multivariate regression analysis, with the disc-fovea angle as dependent variable and all those parameters as independent variables which were significantly associated with the disc-fovea angle in the univariate analysis. From the list of independent parameters we then dropped step by step those parameters which were no longer significantly associated. 95% confidence intervals (CI) were presented. All *P*-values were two-sided and were considered statistically significant if the values were smaller than 0.05.

## Results

Assessable fundus photographs for the determination of the disc-fovea angle were available for 6043 eyes of 3052 (88.0%) individuals with a mean age of 63.6 ± 9.3 years (range: 50 to 91 years) and a mean axial length of 23.2 ± 1.0 mm (range: 18.96–28.87 mm). The group of subjects as compared with the group of individuals without fundus photographs was younger (63.6 ± 9.3 years versus 71.6 ± 10.1 years; *P*<0.001), were less myopic (-0.03, ± 1.64 diopters versus -1.61 ± 3.99 diopters; *P*<0.001), and had a shorter axial length (23.2 ± 1.0 mm versus 23.8 ± 1.9 mm; *P*<0.001). Gender did not vary (*P* = 0.07) significantly between both groups.

**Fig 1:**
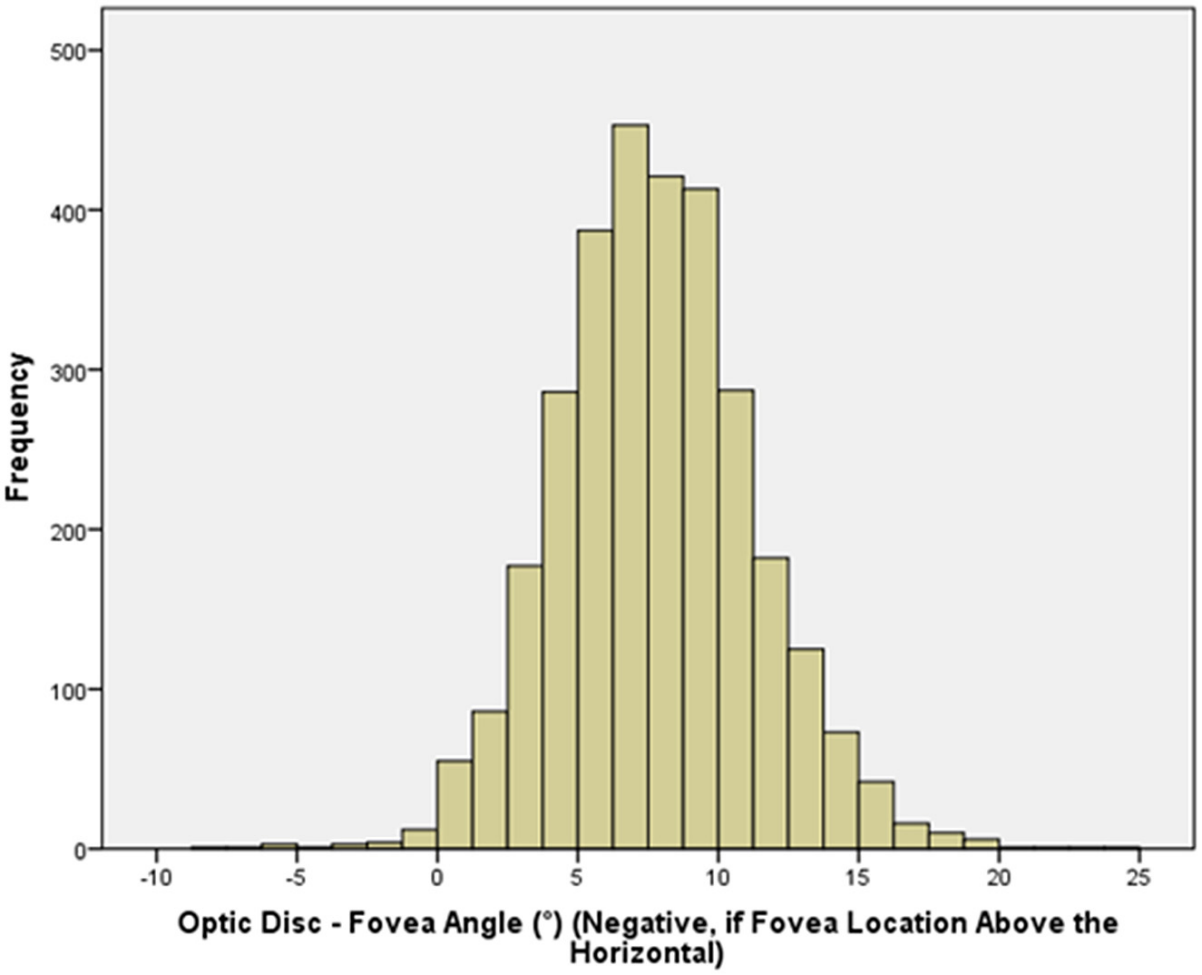
Histogram Showing the Distribution of the Optic Disc—Fovea Angle in the Beijing Eye Study 2011.

Mean disc-fovea angle was 7.76 ± 3.63° (median: 7.65°; range: -6.3° to 28.9°) (Fig 1). It was not normally distributed (*P*<0.001).

In univariate analysis, disc-fovea angle was significantly associated with the systemic parameters of older age (*P*<0.001; r: 0.09), lower body height (*P* = 0.02; r: -0.04), lower body weight (*P* = 0.04; r: -0.04), and with the ocular parameters of higher cylindrical refractive error (*P* = 0.001; r: 0.06), thinner subfoveal choroidal thickness (*P* = 0.005; r: -0.05), larger parapapillary beta zone (*P* = 0.02; r: 0.05), shorter disc-fovea distance (*P* = 0.006; r: -0.05), higher degree of cortical cataract (*P*P = 0.04; r: 0.04), higher prevalence of retinal vein occlusions (*P* = 0.007; r: 0.05), thinner RNFL nasal superior (*P*<0.001; r: -0.15), superior (*P*<0.001; r: -0.13), temporal superior (*P*<0.001; r: -0.14), and temporal (*P* = 0.03; r: -0.04), and thicker RNFL inferior (*P* = 0.02; r: 0.04), nasal inferior (*P* = 0.001; r: 0.06), nasal (*P* = 0.01; r: 0.05), and a higher ratio of inferior RNFL (temporal inferior plus nasal inferior) to superior RNFL thickness (temporal superior plus nasal superior) (*P*<0.001; r: 0.20) (Table 1). The disc-fovea angle was significantly (*P* = 0.01) larger in the group of individuals with a retinal vein occlusion versus the group of participants without retinal vein occlusion (9.25 ± 3.54° (median: 8.7°; range: 0.8° to 19.9°) versus 7.72 ± 3.57° (median: 7.6°; range: -16.6 to 28.9°).

**Table 1:** Associations (Univariate Analysis) between the Disc-Fovea-Angle and Ocular and Systemic Parameters in the Beijing Eye Study 2011.

Parameter	*P*-Value	Standardized Regression Coefficient Beta	Coefficient of Determination	Non-Standard. Regression Coefficient B	95% Confidence Interval of B	Intercept
Age (Years)	<0.001	0.09	0.007	0.03	0.02, 0.05	5.70
Body Height (cm)	0.02	-0.04	0.002	-0.02	-0.04, -0.003	10.9
Body Weight (kg)	0.04	-0.04	0.001	-0.011	-0.023, -0.001	8.53
Cylindrical Refractive Error (Diopters)	0.001	0.06	0.004	0.32	0.14, 0.50	7.54
Subfoveal Choroidal Thickness (μm)	0.005	-0.05	0.003	-0.002	-0.003, 0.001	8.21
Parapapillary Beta Zone Area (mm^2^)	0.02	0.05	0.002	0.21	0.03, 0.38	7.85
Disc-Fovea Distance (mm)	0.006	-0.05	0.003	-0.54	-0.92, -0.15	10.2
Degree of cortical cataract	0.04	0.04	0.002	1.25	0.07, 2.42	7.67
Retinal Vein Occlusion	0.007	0.05	0.002	1.53	0.41, 2.64	7.72
Retinal Nerve Fiber Layer Thickness Nasal Superior Sector (μm)	<0.001	-0.15	0.021	-0.025	-0.031, -0.018	10.3
Retinal Nerve Fiber Layer Thickness Superior Sector (μm)	<0.001	-0.13	0.016	-0.023	-0.029, -0.016	10.6
Retinal Nerve Fiber Layer Thickness Temporal Superior Sector (μm)	<0.001	-0.14	0.020	-0.023	-0.029, -0.017	10.9
Retinal Nerve Fiber Layer Thickness Temporal Sector (μm)	0.03	-0.04	0.002	-0.011	-0.021, -0.001	8.55
Retinal Nerve Fiber Layer Thickness Temporal Inferior Sector (μm)	0.99					
Retinal Nerve Fiber Layer Thickness Inferior Sector (μm)	0.02	0.04	0.002	0.008	0.001, 0.015	6.69
Retinal Nerve Fiber Layer Thickness Nasal Inferior Sector (μm)	0.001	0.06	0.004	0.009	0.004, 0.014	6.69
Retinal Nerve Fiber Layer Thickness Nasal Sector (μm)	0.01	0.05	0.002	0.012	0.002, 0.021	6.93
Ratio of Inferior Retinal Nerve Fiber Layer Thickness (Temporal Inferior Plus Nasal Inferior) to Superior Retinal Nerve Fiber Layer Thickness (Temporal Superior plus Nasal Superior)	<0.001	0.20	0.04	4.02	3.30, 4.74	3.26

The disc-fovea angle was not significantly associated with the systemic parameters of level of gender (*P* = 0.09), region of habitation (*P* = 0.54), waist circumference (*P* = 0.54), level of education (*P* = 0.82), body mass index (*P* = 0.32), systolic blood pressure (*P* = 0.58), diastolic blood pressure (*P* = 0.84), blood concentration of glucose (*P* = 0.51), low-density lipoproteins (*P* = 0.31), triglycerides (*P* = 0.25) and of cholesterol (*P* = 0.30), or with the ocular parameters of central corneal thickness (*P* = 0.83), anterior corneal curvature radius (*P* = 0.33), anterior chamber depth (*P* = 0.054), lens thickness (*P* = 0.07), axial length (*P* = 0.58), refractive error (*P* = 0.56), axis of the cylindrical refractive error (*P* = 0.07), prevalence of refractive high myopia (defined as myopic refractive error of more than -8 diopters) (*P* = 0.47), prevalence of axial high myopia (defined as axial length ≥26.5 mm) (*P* = 0.84), mean RNFL thickness (*P* = 0.053), prevalence of localized retinal nerve fiber layer defects (*P* = 0.06), parapapillary alpha zone (*P* = 0.92), optic disc area (*P* = 0.09), prevalence of early age-related macular degeneration (AMD) (*P* = 0.68), intermediate AMD (*P* = 0.68) or any AMD (*P* = 0.86), degree of nuclear cataract (*P*P = 0.26) and subcapsular posterior cataract (*P*P = 0.99), prevalence of diabetic retinopathy (*P* = 0.20), intraocular pressure (*P* = 0.21), and prevalence of open-angle glaucoma (*P* = 0.63) and angle-closure glaucoma (*P* = 0.94).

The multivariate analysis included the disc-fovea angle as dependent variable. All parameters which were significantly (*P*<0.05) associated with the disc-fovea angle in the univariate analysis were included into the list of independent parameters. Due to reasons of collinearity, we dropped the parameters of ratio of inferior RNFL to superior RNFL thickness (variance inflation factor (VIP): 4.41). Since they were no longer significantly associated with the disc-fovea angle, we then dropped from the list of independent parameters body height (*P* = 0.96), degree of cortical cataract (*P* = 0.99), subfoveal choroidal thickness (*P* = 0.69), area of parapapillary beta zone (*P* = 0.70), disc-fovea distance (*P* = 0.32), RNFL thickness temporal (*P* = 0.60), and body weight (*P* = 0.22).

. In the final model (coefficient of determination r^2^: 0.08), larger disc-fovea angle was associated with older age (*P* = 0.009), thinner RNFL in the nasal superior sector (*P*<0.001), superior sector (*P*<0.001) and temporal superior sector (*P*<0.001) and thicker RNFL in the inferior sector (*P*<001), nasal inferior sector (*P*<0.001) and nasal sector (*P* = 0.007), higher prevalence of retinal vein occlusion (*P* = 0.02), and with larger cylindrical refractive error (*P* = 0.04) (Table 2). If RNFL thickness in the inferior segment was dropped from the model and RNFL thickness in the temporal inferior sector was added, higher RNFL thickness in the temporal inferior segment was associated with a larger disc fovea angle (*P* = 0.002; beta: 0.07). The axis of the cylindrical refractive error was not significantly (*P* = 0.28) associated with the disc-fovea angle if added to the model.

**Table 2:** Associations (Multivariate Analysis) between the Disc-Fovea-Angle and Ocular and Systemic Parameters in the Beijing Eye Study 2011.

Parameter	*P*-Value	Standardized Regression Coefficient Beta	Non-Standardized Regression Coefficient B	95% Confidence Interval of B	Variance Inflation Factor
Prevalence of Retinal Vein Occlusions	0.02	0.04	1.31	0.20, 2.42	1.01
Age (Years)	0.009	0.05	0.02	0.005, 0.02	1.16
Retinal Nerve Fiber Layer Thickness Nasal Superior (μm)	<0.001	-0.17	-0.03	-0.04, -0.02	1.74
Retinal Nerve Fiber Layer Thickness Superior Sector (μm)	<0.001	-0.10	-0.02	-0.03, -0.01	2.23
Retinal Nerve Fiber Layer Thickness Temporal Superior Sector (μm)	<0.001	-0.11	-0.02	-0.02, -0.01	1.60
Retinal Nerve Fiber Layer Thickness Inferior Sector (μm)	<0.001	0.13	0.02	0.01, 0.03	2.35
Retinal Nerve Fiber Layer Thickness Nasal Inferior Sector (μm)	<0.001	0.13	0.02	0.01, 0.03	1.95
Retinal Nerve Fiber Layer Thickness Nasal Sector (μm)	0.007	0.06	0.02	0.004, 0.03	1.41
Cylindrical Refractive Error (Diopters)	0.04	0.04	0.20	0.13, 0.39	1.09

The mean inter-eye difference in the disc-fovea angle expressed as absolute value was 4.01 ± 2.94° (median: 3.49°; range: 0.00–22.3°).

## Discussion

In the sample of our population-based study, the optic disc-fovea angle (mean: 8.71 ± 3.63°) was significantly associated with larger cylindrical refractive error (*P* = 0.04), higher prevalence of retinal vein occlusion (*P* = 0.02), older age (*P* = 0.009), thinner RNFL nasal superior (*P*<0.001) and temporal superior (*P*<0.001), and thicker RNFL nasal inferior (*P*<001) and temporal inferior (*P* = 0.002) (Table 2).

The disc-fovea angle has been addressed in previous investigations [1,3,6.11,14,15,21,23]. Shin et al examined 150 normal children aged 4 to 15 years and a mean disc-fovea angle of 5.13° [23]. In a study by Jethani and colleagues, the disc-fovea angle was measured in 210 eyes of 105 children with an age of 5–15 years [21]. Mean disc-fovea angle was in the right eye 6.49 ± 3.25° and in the left eye 5.80 ± 3.29° without a statistically significant difference between both eyes (*P* = 0.13). Pekel and associates included 90 eyes of 45 healthy individuals with a mean age of 27.3 ± 6.6 years into their study and found a mean disc-fovea angle of 5.24 ± 1.77° in dominant eyes and of 5.49 ± 2.58° in the nondominant eyes without a significant (*P* = 0.51) between both [3]. Rohrschneider measured the disc-fovea angle in 104 healthy individuals with an age ranging between about 20 years and 80 years and found a mean value of 5.6 ± 3.3° [1]. In Rohrschneider’s study as in contrast to our investigation, the disc-fovea angle was not significantly associated with age in univariate analysis. Garway-Heath and colleagues examined RNFL photographs of eyes with normal-pressure glaucoma and found that the position of the optic disc was 15.5 ± 0.9 degrees and 1.9 ± 1.0° above the fovea [6]. These measurements represent a disc-fovea angle of about 6.2°. Amini *et al*. measured the disc-fovea angle on scanning laser ophthalmoscope fundus images from 110 normal eyes and glaucomatous eyes and reported on a mean angle of 6.6 ± 3.4° in the normal eyes and of 7.9 ± 3.9° in the glaucomatous eyes [11]. Using an adaptive optics scanning laser ophthalmoscope in 11 young subjects, Huang et al. measured the angle between the temporal raphe relative to a horizontal line and the raphe-fovea-disc angle (angle between the raphe and the line connecting the disc and fovea center) [5]. The raphe angle was -1.67°±4.8° (range: -9° to 6°), and it was associated with the disc-fovea angle. The raphe-fovea-disc angle was 9.3° ± 3.6°. The values on the disc-fovea angle described above are similar or smaller than the mean value found in our study population with a mean disc-fovea angle of 8.71 ± 3.63°. Reasons for the discrepancy between the studies may be differences in the ethnic background, and to a smaller degree, differences in age.

Previous hospital-based investigations examined the association between the position of the fovea in relationship to the optic nerve head (expressed as the disc-fovea angle) and the regional distribution of the RNFL thickness or the functional-structural diagnosis of glaucoma. Choi and colleagues examined 164 healthy myopic individuals and found that a larger disc-fovea angle was significantly associated with a decreasing superior RNFL thickness, an increasing inferior RNFL thickness and subsequently with an increasing inferior-to-superior RNFL thickness difference [14]. The authors concluded that the disc-fovea angle characterizing the position of the fovea in relationship to the optic disc was an important determinant of the normal pattern of the RNFL thickness. Investigating the rate and associated factors of false-positive diagnostic classification of ganglion cell analysis and retinal nerve fiber layer maps obtained by optical coherence tomography, Kim and colleagues examined 104 normal individuals and found that a false-positive ganglion cell analysis diagnostic classification was associated with a larger fovea-disc angle [17]. Amini *et al*. reported that adjusting for the disc-fovea angle improved the prediction of glaucoma by the RNFL assessment for some fundus sectors [11]. Lamparter and colleagues found that the position of the optic nerve head in relation to the fovea was the most prominent predictor for variations in the mapping of retinal locations of visual field to the optic nerve head, followed by other parameters such as disc area, axial length, refractive error, disc shape, disc orientation, and disc tilt [10]. The model proposed by Denniss and colleagues predicted that changes in the position of the optic disc resulted in retinal nerve fibers being distributed differently around the disc [8,12,13]. For example, moving the disc superiorly resulted in more fibers entering the inferior portion of the disc, which matched the results of the present study. Similar results could be obtained applying the model described by Jansonius and colleagues [9]. Asaoka and colleagues reported that a new visual field test grid with fewer test locations and centered at the optic disc had a stronger structure-function correlation than the conventional 24–2 visual field test [7]. In a similar manner, Garway-Heath *et al*. found that the position of the optic nerve head had a significant effect on the corresponding position of test positions in the visual field [6]. All these studies including our investigation agree upon that the disc-fovea angle influences the pattern of the RNFL thickness. It may therefore be taken into account if the RNFL thickness pattern is used for a morphological detection of optic nerve diseases including glaucoma.

The association between a larger disc-fovea angle and a higher cylindrical refractive error corresponds to the association between higher corneal astigmatism and a more oblique optic disc shape [32]. It supports the notion of an association between an abnormally shaped anterior segment (i.e. increased corneal astigmatism) and an abnormally shaped posterior segment.

Interestingly, the disc-fovea angle was not significantly associated with axial length. It may indicate that the axial elongation associated changes including thinning of the sclera and choroid, enlargement of the optic nerve head with subsequent thinning of the lamina cribrosa and peripapillary scleral flange, and the development of myopic maculopathy including occurrence of macular Bruch’s membrane defects, do not include a change in the disc-fovea angle [24,25,26]. One may infer that the marked axial myopia associated alterations at the posterior pole occur in a geometrically symmetrical manner in the sense that the orientation between fovea and optic nerve head is not affected.

The clinically most important association between the disc-fovea angle and other parameters may be the relationship between a larger disc-fovea angle and thinner RNFL superiorly and thicker RNFL inferiorly (Table 2). These findings obtained in a population-based survey in a multivariate analysis agree with the observations made in hospital-based studies mentioned above. It may show the clinical importance of the disc-fovea angle and may warrant getting it automatically measured by imaging devices and included into algorithms for the morphologic diagnosis of glaucomatous optic neuropathy. It may further improve the structural diagnosis of glaucoma by the assessment of the RNFL. Although the neuroretinal rim width was not measured in this study, one may discuss that also the rim shape depends on the position of the fovea in relationship to the optic disc (i.e., the disc-fovea angle). One may infer that in eyes with a large disc-fovea angle as compared to eyes with a small disc-fovea angle the inferior rim may be relatively wider as compared to the superior rim. Taking the disc-fovea angle into account when assessing the neuroretinal rim shape may thus increase the diagnostic precision of the so called ISNT (Inferior-Superior-Nasal-Temporal)-rule [33]. The inclusion of the disc-fovea angle may also increase the diagnostic value of localized RNFL defects as determined by OCT, since the regional distribution of the thickness of the RNFL depends on the disc-fovea angle. Eyes with an abnormal disc-fovea angle could have a normal RNFL, the regional distribution of which would be abnormal but physiological, parallel to the abnormal position of the fovea. If the abnormal fovea position is not taken into account, the contour line of the RNFL thickness as measured by OCT would then fall into the red region on the RNFL thickness print-out and could thus erroneously be considered to be pathological.

The association between a larger disc-fovea angle and a higher prevalence of retinal vein occlusions has remained elusive and future studies may examine whether this observation was co-incidental or may point to a structural risk factor of a large disc-fovea angle for an increased prevalence of retinal vein occlusions.

Potential weaknesses of our study should be discussed. First, fundus photographs were assessed for 88.0% of the overall study participants, and the original participation rate of all eligible subjects in the Beijing Eye Study was 78.8%. Although the group of participants differed from the group of non-participants in age, refractive error and axial length, one may assume that the participation rate was sufficient to allow conclusions on normative values such as the disc-fovea angle and its associations. Second, the minimal age in our study was 50 years so that the observations made in our study cannot directly be transferred on younger individuals. Third, the present study included Chinese individuals. Since ocular dimensions may differ between ethnicities, the measurements obtained in our study population may not directly be transferred onto other populations. Fourth, since we measured the disc-foveal angle using fundus photographs, any head torsion influenced the measurements as also examined and discussed by Denniss and colleagues [13]. Although the study participants were asked to keep their head in a vertical position and although the head position was checked by the examiner, minor head torsions will have had an impact on the orientation of the fundus photographs and thus on the measurements of the disc-fovea angle. Since however, a head torsion will have occurred in a random manner, this weakness of the method will have increased the noise of the examination, but may not have markedly influenced the results and conclusions of the study. Fifth, although the associations in the final model of the multivariate analysis were statistically significant, the model accounted only for approximately 8% of the variance in the disc-fovea angle. This relative weakness of the association holds true for the multivariate analysis as well as for the univariate associations (Table 1). Sixth, we tested many parameters in the univariate analysis for an association with the disc-fovea angle, including those variables that primarily might not have been expected to be reasonably associated with the disc-fovea angle. It refers to variables such as the region of habitation or the level of education. These variables however, were strongly correlated with axial length and myopia which were some of the key parameters to be tested for associations with the disc-fovea angle. We therefore included a whole list of parameters into the univariate analysis in the first step of the statistical analysis in an attempt not to overlook a potentially confounding effect by some of these parameters.

In conclusion, the optic disc-fovea angle markedly influences the regional distribution of the RNFL thickness pattern. The disc-fovea angle may routinely be taken into account in the morphological glaucoma diagnosis and in the assessment of structure-function relationship in optic nerve diseases. Future studies may address potential associations between a larger disc-fovea angle and retinal vein occlusions and between the disc-fovea angle and the neuroretinal rim shape.
